# Social and Behavioural Change Communication Challenges, Opportunities and Lessons from Past Public Health Emergencies and Disease Outbreaks: A Scoping Review

**DOI:** 10.5334/aogh.4418

**Published:** 2024-10-23

**Authors:** Laston Gonah, Sibusiso Cyprian Nomatshila

**Affiliations:** 1 Department of Public Health, Faculty of Medicine and Health Sciences, Walter Sisulu University, South Africa

**Keywords:** Social Behavioural Change (Communication), Public health emergencies, Ebola, COVID-19, Monkeypox, Cholera

## Abstract

*Background:* Documentation of social behavioural change communication (SBCC) regarding challenges, opportunities and lessons drawn from past public health emergencies is worthwhile to inform priorities for future response efforts.

*Aim:* The aim of this review is to scope the evidence on social behavioural change communication regarding challenges, opportunities and lessons drawn from Ebola, coronavirus disease 2019 (COVID-19), monkeypox and cholera outbreaks from studies published before March 2024, and suggest priorities for future response efforts.

*Methods:* A Boolean strategy was used to search electronic databases for relevant published articles, complemented by relevant studies identified from reference lists.

*Results:* The challenges, opportunities, lessons learnt and priorities for SBCC were consistent across study contexts, showing marked variations over time. The significance of technology, infodemic management, and behavioural data generation emerged more frequently and became increasingly important over time. Identified challenges were uptake hesitancy, limited capacity to undertake infodemic management, inadequate funding and human resources for SBCC, competing priorities, parallel or conflicting interventions due to inadequate coordination, difficulties evaluating SBCC programmes and missed opportunities for integration into routine programmes. Existing supportive structures for SBCC, strong political will and participation, as well as rapid information exchange enabled by technological advancement, represented opportunities for enhancing the effectiveness of SBCC programmes. Key lessons were that a multisectoral approach and coordination, partnership and active collaboration amongst stakeholders; building/strengthening trust, target population segmentation and localization of interventions, are important for enhancing the effectiveness of SBCC programmes. Political will, involvement and participation represent the core of social behavioural change (communication) interventions during a public health emergency.

*Conclusion:* SBCC programming for future response to public health emergencies and disease outbreaks should consider the diverse assortment of benefits, threats/challenges and opportunities brought about by technology, infodemics and behavioural data generation to be more effective.

## Introduction

Social and behavioural change communication (SBCC) was shown to be a key component of past response efforts in public health emergencies and disease outbreaks such as Ebola, coronavirus disease 2019 (COVID‑19), monkeypox and cholera [1–3]. SBCC refers to all information exchange efforts aimed at promoting informed compliance with recommended measures or promoting positive behaviour change required to interrupt a public health emergency [2]. The communication efforts involve creating supporting environments to enhance information exchange and promote behaviour change; use of behavioural data to identify information needs; and to make the needed information relevant in terms of language, medium, content and changing pandemic phases [4, 5]. The interruption of Ebola, COVID‑19, monkeypox, cholera and other outbreaks has always depended on compliance with recommended measures mainly in the form of positive behaviour change; hence, there are SBCC challenges revealed, opportunities presented and lessons learnt as a result of each of these past public health emergencies that can be useful in setting priorities for future outbreaks.

Since the first outbreak, several changes have occurred, such as population mobility patterns, technology and sociocultural changes, that may have impacted the art and science of SBCC in various ways [1, 4, 5]. Documenting SBCC lessons learnt from past public health emergencies and disease outbreaks is worthwhile to inform SBCC programming for future responses to emergencies and disease outbreaks. This exercise is important for exploring the trends, patterns and relationships existing in SBCC as a complex problem, and for using this information to predict/anticipate challenges and opportunities in setting priorities for future SBCC strategies. The paper sought to scope the evidence on social behavioural change communication regarding challenges, opportunities and lessons drawn from Ebola, COVID‑19, monkeypox and cholera outbreaks from studies published before March 2024, and suggest priorities for future response efforts.

## Methods

### The search strategy

A scoping review of relevant literature was conducted using PubMed, PubMed Central, United Nations (UN) agencies websites [UN International Children's Emergency Fund (UNICEF) and World Health Organization (WHO)], African Journals Online, Embase and Google Scholar, complimented by relevant additional articles identified from reference lists of the selected publications. The formulation of study questions and search syntax was informed by a modified population/patient, intervention/variable, comparison, outcome, time and setting/context (PICOTS) framework, presented in Table 1 (Samson 2012) [6].

**Table 1:** Modified population/patient, intervention/variable, comparison, outcome, time and setting/context (PICOTS) framework used in search syntax formulation.

POPULATION/PATIENT	PEOPLE AFFECTED BY EBOLA, COVID‑19, MONKEYPOX AND CHOLERA OUTBREAKS, OR INVOLVED IN THE RESPONSE
**I**ntervention/variables	Social behavioural change (communication) interventions or campaigns; key messages
**C**omparison	People not affected by Ebola, COVID‑19, monkeypox or cholera outbreaks; previous outbreaks; strategies employed in other settings
**O**utcome	Observed behaviours; identified SBCC challenges, opportunities, lessons learnt, priorities and recommendations
**T**ime	Studies conducted and published before March 2024
**S**etting/context	Worldwide (low‑, lower‑middle‑, upper‑middle‑ and high‑income countries)

Source: Samson D, et al. (2012) [6].

Key search terms included were the following, in various combinations: (Social and behavioural change OR communication OR messages OR Interventions OR Strategies OR Campaigns AND knowledge, attitudes, skills, beliefs and social norms) AND (lessons learnt OR challenges OR opportunities OR Priorities) AND (from past public health emergencies and disease outbreaks) OR (Ebola, Cholera, COVID‑19, monkeypox).

### Inclusion and exclusion criteria

The review only included studies written in the English language and published before March 2024. Selected studies should have focused on at least any one of the categories SBCC challenges, SBCC opportunities, SBCC lessons learnt or SBCC future priorities/recommendations for any one of Ebola, COVID‑19, Monkeypox and cholera.

Studies focusing on patient populations other than those affected by Ebola, COVID‑19, monkeypox and cholera were excluded. Opinion papers, supplement letters, letters to the editor, abstracts, preprints, review articles and viewpoints were not included in this review.

### Methodological appraisal/scoring method for review articles

**Figure 1:**
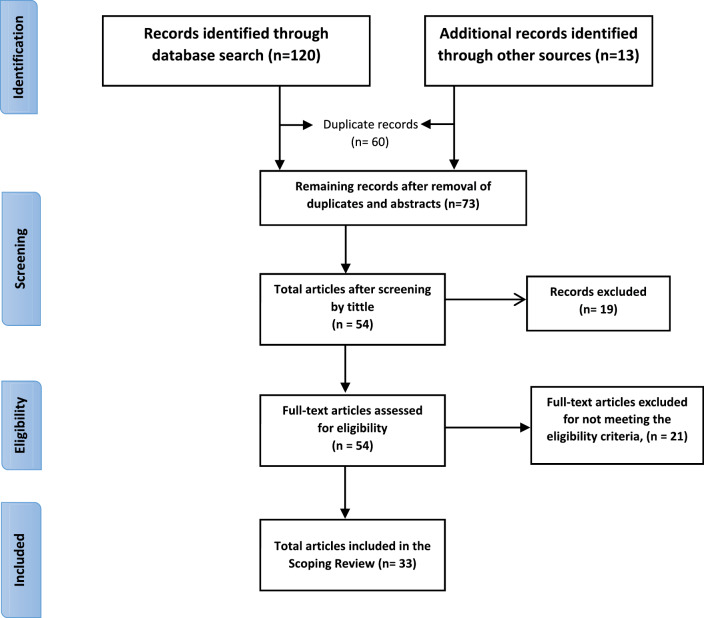
Preferred Reporting Items for Systematic Reviews and Meta‑analyses (PRISMA) flow diagram summarizing the search process and results selection. Adapted from Moher D, et al. (2009) [8].

An adapted six‑item quality assessment tool for systematic reviews of observational studies [7] (QATSO) was used to assess the quality of each of the identified articles. The six items considered in determining article quality are (1) the representativeness of the sampling method used, (2) whether the study statistically determined the sample size or whether the sample size gave the study adequate power, (3) whether the study clearly stated the eligibility criteria, (4) whether the outcomes measures were objectively ascertained as opposed to subjective outcome ascertainment, (5) whether the study clearly determined or assessed the outcome measures and (6) whether the study employed any strategies to control for potential bias or confounding (e.g. by stratification, matching or use of controls) in at least one of study design or data analysis [7]. Each of the six items could be answered by either a “Yes” or a “NO,” where a “Yes” carried a score of 1 point and a “No” scored 0 points, giving a maximum possible score of 6 points and a minimum possible score of 0 points, per article. Articles/studies rated as poor or scoring below 3 points out of 6 were excluded from the final analysis, resulting in 33 articles being included (Table 2 and Figure 1).

**Table 2:** Scoring method for review articles.

GRADING	5 OR 6 OUT OF 6	3 OR 4 OUT OF 6	0, 1 OR 2 OUT OF 6
Risk of bias	Low	Medium	High
Study quality	Good	Satisfactory	Poor
Number of articles identified (*n* = 54)	21 (38.9%)	12 (22.2%)	21 (38.9%)

Adapted from Wong et al. (2008) [7].

The Preferred Reporting Items for Systematic Reviews and Meta‑analyses (PRISMA) guided the scoping review. The PRISMA statement, used in Figure 1, gives a minimum set of items to guide the reporting of systematic reviews and meta‑analyses.

## Results

### Description of the selected study articles

A total of 33 articles meeting the eligibility and inclusion criteria were identified and included in the final analysis. Most of the studies (17) were conducted in sub‑Saharan Africa, and the remaining ones in America (7), Europe (6) and Asia (3). All but one study employed a non‑experimental cross‑sectional design, and one of them compared models of individual and collective learning in regard to risk perception and behavioural change [9]. The studies were aimed at various aspects of public health emergencies, addressing at least one of the challenges, lessons learnt, opportunities and priorities for at least one of the target disease outbreaks. Findings from the review were categorized as challenges, opportunities, key lessons learnt and suggested SBCC priorities.

### Challenges

There was consistency in findings, concerning SBCC challenges, from all study contexts. However, issues related to infodemics frequently emerged in articles published after 2019 [10–18]. Several challenges were identified that were faced in responding to public health emergencies and disease outbreaks:

- Uptake hesitancy and resistance
  Compliance with recommended public health control measures was generally found to be hindered by uptake hesitancy and resistance, mostly driven by fears, doubts/lack of trust, fatigue, beliefs, politicization of response initiatives and lack of knowledge [4, 15, 17, 19, 20]. There was a commonality of findings across health topics and study contexts showing that hesitancy and resistance to comply with recommended public health measures is driven by fear of adverse events following uptake of recommended medical products; mistrust of healthcare workers’ advice or doubts over the effectiveness of recommended public health measures; counterproductive religious or cultural beliefs shaping perceived disease causality and treatment options; fatigue or waning motivation to comply with recommended measures due to prolonged emergencies or outbreaks; and lack of knowledge or awareness of disease causality, spread, preventive and treatment measures [1, 4, 17, 18].
  Addressing these uptake barriers is believed to result in improved uptake. Factors such as trust in healthcare workers’ advice; perceived or observed vaccine efficacy; motivation from a role model/political/religious/traditional leader; government/political will and participation in the response; correct knowledge about disease causes, prevention and management; and having the skills and resources needed to prevent, treat and manage the diseases were consistently found to be important in improving uptake and compliance with recommended response measures across study contexts and health topics [1, 4, 19, 21–23].
- Infodemic management
  The widespread use of the internet as a source of information lead to an excessive amount of information, including false and/or misleading information being spread during public health emergencies and disease outbreaks [1, 4, 10, 18, 24]. Identified challenges included insufficient financial resources and limited human resource capacity for the implementation of infodemic management, especially for undertaking social listening, feedback collection, media research, media or rumour monitoring, myth‑busting and e‑health literacy promotion at subnational levels [4, 10, 15, 18]. The findings were consistent across health topics and study contexts, and mentioned more frequently in studies conducted after 2019. It was also observed that much of the false information that influence public behaviour is spread by trusted and influential people, both online and offline [5, 10, 16, 18, 25]. This implies that involvement of trusted and influential people in the design of behavioural change and communication strategies and materials is worthwhile when it comes to addressing the counterproductive effect of infodemics during a health emergency. Moreover, credible and trusted sources of information must ensure that they are up to date with prevailing rumours and public concerns by putting in place efficient mechanisms for behavioural data generation and must ensure that the necessary information is updated in time, to align with the rumours and concerns [4, 5].
- Inadequate funding and human resources for SBCC or SBCC being less prioritized
  Across reviewed literature, there was commonality in findings that social behaviour change communication (SBCC) programmes are understaffed and less prioritized compared with other response pillars during public health emergencies and disease outbreaks [3, 9, 26, 27]. SBCC encompasses multiple technical fields/specialities wherein the skills may not be possessed by one individual, such as risk communication and community engagement (RCCE), advocacy, policy analysis and design, graphic design and media monitoring, amongst others [4, 9]. Inadequate funding and understaffing of SBCC programmes were found to lead to some specialities being underrepresented, having only one person who had multiple responsibilities or who was working in more than one technical area, or some technical areas not being represented at all, hence undermining programme effectiveness in emergency response [9, 28, 29]. This can be worsened by the possible co‑occurrence of public health emergencies and humanitarian crises naturally demanding more personnel, such as the co‑occurrence of COVID‑19, monkeypox and humanitarian crises in the Horn of Africa in 2022 [4, 9, 28, 29]. It is recommendable that staffing decisions are informed by determination and consideration of all the SBCC‑related workload indicators of staffing need (WISN) to ensure that all response pillars are equally prioritized [9].
- Competing priorities, e.g., public health emergencies, other humanitarian crises and work–life balance
  As has been witnessed before, there is a possibility that multiple public health emergencies can occur at the same time, as was observed with COVID‑19, polio, monkeypox and humanitarian crises in the Horn of Africa in 2022 [9, 16, 17, 27, 30, 31]. This results in competing priorities, as resources (financial, material and human) need to be distributed wisely to address the co‑occurring public health emergencies and humanitarian crises. Emerged negative effects on SBCC programmes were excessive workload and inadequate resources, compounded by staff burnout/fatigue due to negative work–life balance, consequently resulting in ineffective response [4, 9].
- Parallel or conflicting interventions due to inadequate partner coordination
  The existence of parallel programmes emerged as a common challenge during public health emergencies, usually emanating from different funding sources and the response focus of the participating organizations or stakeholders [4, 5, 24]. While it may seem positive to have many organizations supporting the response, this is usually associated with many challenges for SBCC if not properly coordinated. There was convergence of evidence in research findings across health topics and study contexts that the existence of uncoordinated parallel programmes usually results in replication of response activities, leading to inefficient use of resources [1, 3, 5, 29, 32]. In SBCC, parallel programmes involving dissemination of conflicting messages/advice by different organizations were also found to be one of the factors responsible for causing public doubt or mistrust in advice from healthcare workers, leading to noncompliance or resistance [16, 19, 24, 33], whereas coordinated SBCC programmes, involving multisectoral collaboration in strategy design, implementation and evaluation were found to contribute to the success of SBCC programmes, in addition to the cost‑effectiveness advantage [5, 25, 27].
- The difficulty of evaluating SBCC through randomised controlled trials
  Monitoring and evaluation represent a key component for determining whether a programme is on track to achieve the set goals. While other public health emergency response pillars track their progress using some set of standard quantitative indicators or “hard” clinical endpoints, evaluating the net contribution of SBCC programmes when it comes to behaviour change and diseases outcome was reported to be a challenge [2, 4, 5, 12, 34]. SBCC programmes must invest more in strengthening behavioural data generation and establishing standard ways of monitoring and evaluating their activities.
- Missed opportunities for integration into routine programmes
  Despite the co‑occurrence of public health emergencies and humanitarian crises, SBCC response activities for past emergencies were found to be largely parallel, with each disease‑specific health event or humanitarian crisis having its own response strategy, yet sometimes targeting the same audience/population [1, 16, 24, 27]. This situation was found to cause an additional burden to the intended programme recipients, as the same population may sometimes be expected to make time for participating in different programmes at conflicting times, when the emergency response programme could have been integrated into routine programmes [23]. This burden may end up confusing the targeted population, as they are flooded with different information, leading to non‑compliance, resistance and little participation in response initiatives [5, 9]. In SBCC, non‑integration into routine programmes results in too much information that is difficult to adhere to, for instance, SBCC for COVID‑19, for monkeypox, for cholera and for polio, together with that for routine programmes such as human immunodeficiency virus (HIV)/acquired immune deficiency syndrome (AIDS), tuberculosis (TB) and non‑communicable diseases NCDs. Response to past public health emergencies was found to have overemphasized responding to new health events and missed the opportunity for integrating the emergency response initiatives into routine public health programmes [28, 29]. Future preparedness and emergency response initiatives must consider the feasibility of ensuring uninterrupted continuity of routine healthcare service provision whilst responding to pandemics through the integration of emergency response initiatives into routine programmes.

### Opportunities

Existing supportive community, government and political structures and partners for SBCC emerged as key opportunities that can be leveraged for ensuring swift and effective response to future health events [5, 9, 28, 29]. The readiness and rich experience in SBCC that these existing structures possess were found to be key in ensuring timely and efficient response to future health threats, and this consistently emerged across study contexts, health topics and time periods [4, 28, 35]. These structures are also known to be the same structures that support routine programmes, hence making integration more technically efficient and cost‑effective.

SBCC was found to be more feasible in countries where there was political will to address emergencies or disease outbreaks [5, 28, 36]. For instance, in countries where the leadership had resisted the COVID‑19 vaccine rollout, SBCC programmes for vaccine uptake were considered illegal or given little priority [19, 25]. In those countries where there was no political will and commitment, there was little to no buy‑in from stakeholders, consequently leading to limited/non‑enforcement of behaviour change and poor/low uptake or resistance, even in the presence of SBCC programmes [1, 26, 32, 36]. SBCC alone as a technical approach is less likely to achieve the intended goals without political will and support. Actual behaviour change happens when the political leadership owns the response, leading to buy‑in from stakeholders and the targeted population. Political will has the power and capacity to commit the resources and necessary policies required to support the SBCC strategy.

Technological advancement has enabled fast information exchange, for instance, through chat boards, bulk SMS and social media [4, 10, 11, 18, 24, 35]. While this may present a threat for the spread of false information, there was consensus amongst study findings that these channels can be used for credible information exchange that can stand out against infodemics if transparently, consistently and timeously updated to address people’s concerns, needs and fears. The role of technology in SBCC has become increasingly important and an often‑mentioned issue in studies published after 2019, across health topics and contexts [4, 11, 15, 18]. Undoubtedly, technology brings a diverse assortment of threats and benefits, requiring a cautious and well‑planned approach to utilizing and managing it.

### Key lessons learnt

1. As discussed before, political will, involvement and participation represent the core of social behavioural change (communication) interventions during a public health emergency (Figure 2).

**Figure 2:**
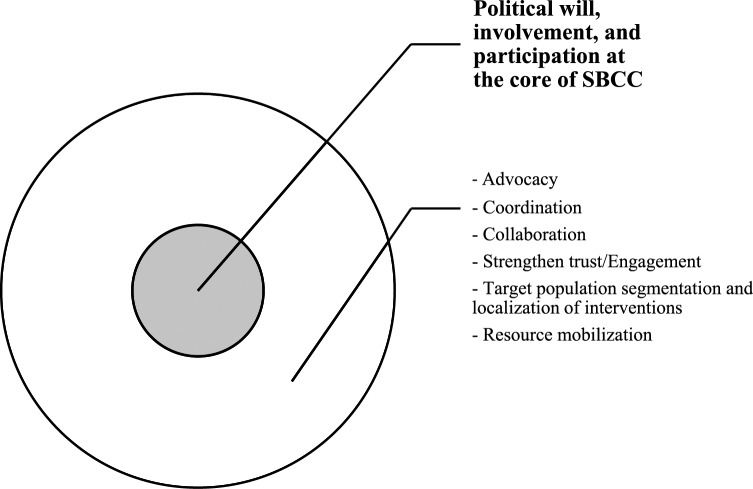
Key factors for successful SBCC interventions during a public health emergency.
  When no public health emergencies are occurring, response efforts are usually healthcare providers’ remit. However, when a public health emergency strikes, public attention abruptly shifts to political leadership for countermeasures and guidance [9, 28]. In other words, when “things go really bad” or when an emergency comes up that threatens people’s lives and wellbeing, the government/political leadership usually automatically and immediately steps in and assumes responsibility, ahead of all response actors. The public health threat, if big enough, automatically draws the attention of political leadership, putting them at the forefront of response efforts, as observed during Ebola and COVID‑19 outbreaks [16, 28, 29]. Political leadership plays a key role in resource mobilization, as well as influencing and enforcing behaviour change by the affected population [4, 16, 28]. This demonstrates the inextricable link between public health, security and politics. Therefore, a public health technical taskforce should be swift enough in perceiving an imminent health threat and in the determination of its potential impact on socio‑economic wellbeing, disease severity, injury/long‑term impairments and mortality in the affected population, and in bringing this picture to the attention of political leadership to make appropriate decisions, contributions and/or declarations. Therefore, SBCC interventions in emergency preparedness and response require strong and sustained partnership and collaboration between political leadership and technical stakeholders in ending the public health threat.
2. A multisectoral approach and coordination are key for ensuring the effectiveness of response efforts.
  Successful SBCC approaches were found to depend on strong partnership between stakeholders, clear delineation of partner roles and coordination of response activities for a swift and efficient response, and this consistently emerged from study findings across health topics and study contexts [2, 4, 5]. Future SBCC initiatives are encouraged to prioritize undertaking stakeholder mapping and engagement, co‑constructing SBCC strategy with all stakeholders and ensuring transparency and consistency on feedback mechanisms and information sharing.
3. It also consistently emerged across health topics and study contexts that partnership and active collaboration amongst all stakeholders, including with the community, political leadership, non‑state actors (NSA) and civil society organizations (CSOs), is critical to enhance resource pooling for SBCC interventions; promote ownership of and full participation in response efforts; and strengthen collaboration and widening coverage of SBCC programmes [5, 16, 22, 27, 37, 38]. This requires periodic review meetings for reflection, identification of synergies and planning.
4. Engagement of trusted/influential people or institutions, for example, community and religious leaders, socialites and politicians, emerged as essential for promoting and sustaining behaviour change [3, 27, 38]. Behaviour change and compliance with recommended public health measures is based on the perceived effectiveness of proposed measures, which is rooted in trust in the sources of information.
5. Segmenting the target population, contextualizing/localizing interventions and revising/reviewing SBCC material according to changing pandemic situation/needs was seen to be more effective in addressing information needs, improving health literacy and promoting positive behaviour change [4, 9, 38].
6. Future SBCC initiatives should prioritize resource mobilization and advocacy for increased resource allocation to sustain implementation of SBCC programmes [5, 29, 32, 38]. SBCC and risk communication are usually regarded as the least amongst public health emergency response pillars, and hence are least prioritized when it comes to resource allocation [4, 5]. Efforts should be made to improve behavioural data generation and monitoring and evaluation of SBCC initiatives to demonstrate its importance and justify the need for increased resource allocation towards SBCC programmes.

### Suggested SBCC priorities or strategies

1. Evidence or behavioural data generation for data‑driven SBCC interventions throughout the emergency or disease outbreak phase is important, including putting in place standard measures for evaluating SBCC programmes.
2. Consider an integrated and well‑collaborated approach to managing public health emergencies and disease outbreaks in a balanced partnership with communities. This includes integrating the emergency interventions into routine or essential health programmes.
3. Strengthen infodemic management, community feedback mechanisms, documentation, and monitoring and evaluation for SBCCs, including capacity‑building on these, at all levels.
4. Find ways of strengthening advocacy for active involvement and participation of political leaders and stakeholders to revitalize the response.
5. Support national and sub‑national structures in strengthening SBCC capacity, especially in regions showing poor disease outcomes.

## Summary

**Figure 3:**
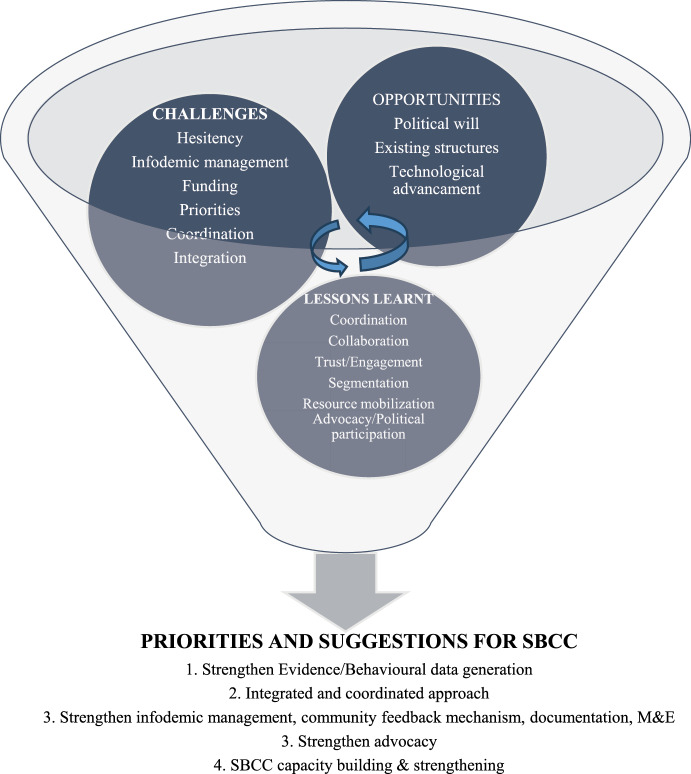
Interaction of challenges, priorities, opportunities, lessons learnt and priorities and suggestions for SBCC.

The challenges, priorities, opportunities, lessons learnt and suggested priorities for SBCC are summarized in Figure 3 below.

## Conclusion

The challenges, opportunities, lessons learnt and priorities for SBCC were consistent across study contexts, showing marked variations over time. While no marked variations were noted in regard to other identified SBCC challenges, opportunities, lessons learnt and priorities across contexts, the significance of technology in SBCC, infodemic management and behavioural data generation for data‑driven SBCC interventions emerged more frequently and became increasingly important over time. SBCC programming for future response to public health emergencies and disease outbreaks should consider the diverse assortment of benefits, threats/ challenges and opportunities brought about by technology, infodemics and behavioural data generation to be more effective.
